# Targeting ferroptosis: a novel therapeutic strategy for the treatment of retinal diseases

**DOI:** 10.3389/fphar.2024.1489877

**Published:** 2024-10-30

**Authors:** Xiao-Dan Hao, Wen-Hua Xu, Xiaoping Zhang, Junqiang Xue

**Affiliations:** ^1^ Department of Rehabilitation Medicine, The Affiliated Hospital of Qingdao University, Qingdao, Shandong, China; ^2^ Institute for Translational Medicine, The Affiliated Hospital of Qingdao University, College of Medicine, Qingdao University, Qingdao, China; ^3^ Institute of Regenerative Medicine and Laboratory Technology Innovation, Qingdao University, Qingdao, Shandong, China; ^4^ Department of Ophthalmology, The Affiliated Hospital of Qingdao University, Qingdao, Shandong, China

**Keywords:** ferroptosis, retinal diseases, regulatory mechanism, ferroptosis targeting agents, ferroptosis inhibitors

## Abstract

Ferroptosis plays a vital role in the progression of various retinal diseases. The analysis of the mechanism of retinal cell ferroptosis has brought new targeted strategies for treating retinal vascular diseases, retinal degeneration and retinal nerve diseases, and is also a major scientific issue in the field of ferroptosis. In this review, we summarized results from currently available *in vivo* and *in vitro* studies of multiple eye disease models, clarified the pathological role and molecular mechanism of ferroptosis in retinal diseases, summed up the existing pharmacological agents targeting ferroptosis in retinal diseases as well as highlighting where future research efforts should be directed for the application of ferroptosis targeting agents. This review indicates that ferroptosis of retinal cells is involved in the progression of age-related/inherited macular degeneration, blue light-induced retinal degeneration, glaucoma, diabetic retinopathy, and retinal damage caused by retinal ischemia-reperfusion via multiple molecular mechanisms. Nearly 20 agents or extracts, including iron chelators and transporters, antioxidants, pharmacodynamic elements from traditional Chinese medicine, ferroptosis-related protein inhibitors, and neuroprotective agents, have a remissioning effect on retinal disease in animal models via ferroptosis inhibition. However, just a limited number of agents have received approval or are undergoing clinical trials for conditions such as iron overload-related diseases. The application of most ferroptosis-targeting agents in retinal diseases is still in the preclinical stage, and there are no clinical trials yet. Future research should focus on the development of more potent ferroptosis inhibitors, improved drug properties, and ideally clinical testing related to retinal diseases.

## 1 Introduction

Diseases of the retina are a primary factor in causing permanent blindness and vision loss, impacting millions of individuals globally (Hoon et al., 2014). Retinopathy has many classifications that can affect retinal blood vessels, nerves and cells, including age-related macular degeneration (AMD), central retinal artery occlusion and branch artery occlusion, central retinal vein occlusion and branch vein occlusion, retinal detachment, diabetic retinopathy (DR), retinitis pigmentosa (RP), retinal tumor, congenital eye disease and intraocular parasites (Hoon et al., 2014; Mead and Tomarev, 2020). Retinopathy can lead to vision loss, macular edema, retinal detachment, fundus hemorrhage, visual field defects, and in severe cases, blindness, which seriously affects the quality of life of patients. Studying the pathogenesis of retinal diseases and exploring potential treatment strategies are hot spots and difficulties in this field (Kaji et al., 2018; Mead and Tomarev, 2020; Dhurandhar et al., 2021).

Ferroptosis is a programmed death modality of non-apoptotic cells that relies on the accumulation of iron in cells, resulting in an increase in the toxic lipid peroxide reactive oxygen species (ROS) (Jiang et al., 2021). Lipid peroxidation of unsaturated fatty acids that are highly expressed on the cell membrane in response to ferric iron or esteroxygenase induces cell death (Rochette et al., 2022). The characteristics of ferroptosis cells mainly include smaller mitochondria, increased membrane density, decreased cristae, increased intracellular lipid peroxidation, increased ROS, and iron ion aggregation (Jiang et al., 2021; Tang D. et al., 2021). In addition, ferroptosis is also manifested by a decrease in protein GPX4 levels, the core enzyme that regulates the antioxidant system (glutathione system) (Jiang et al., 2021; Tang D. et al., 2021; Li F. et al., 2023).

Iron is essential for retinal metabolism, but an excess of ferrous iron causes oxidative stress, which is one of the main mechanisms for the occurrence of ferroptosis (Youale et al., 2022). Iron overload is essential for ferroptosis execution (Liu K. et al., 2023). Under physiological conditions, circulating ferric irons (Fe^3+^) that bind to transferrin (TF) are transported into cells via transferrin receptor (TFRC). Inside the cells, Fe^3+^ undergoes reduction to ferrous (Fe^2+^) and is then exported by divalent metal transporter one in the cytosol, contributing to the labile iron pool. Excess intracellular free Fe^2+^ facilitates the reduction of oxygen, generating superoxide radicals, and causes lipid peroxides through Fenton reactions, resulting in ferroptosis finally (Liu K. et al., 2023). Several factors, including hypoxia, overload of all-transetinal (atRAL), high intraocular pressure, aging, and iron metabolism imbalance, lead to abnormal accumulation of iron in cells and retinas by affecting the proteins involved in the iron metabolism and homeostasis (Ugarte et al., 2013; Chen et al., 2021; Vogt et al., 2021; Henning et al., 2022; Yao et al., 2023).

Recently, many studies have shown that ferroptosis plays a vital role in the progression of various retinal diseases, and targeting ferroptosis provides promising potential strategies for the treatment of ferroptosis-related retinal diseases. The analysis of the mechanism of retinal cell ferroptosis has brought new-targeted strategies for the treatment of retinal vascular diseases, retinal degeneration and retinal nerve diseases, and is also a major scientific issue in the field of ferroptosis. In this review, we summarized results from currently available *in vivo* and *in vitro* studies of multiple eye disease models, clarified the pathological role and molecular mechanism of ferroptosis in retinal diseases, summed up the existing pharmacological agents targeting ferroptosis in retinal diseases as well as highlighting where future research efforts should be directed for the application of ferroptosis targeting agents.

## 2 Pathological role of ferroptosis in retinal diseases

**FIGURE 1:**
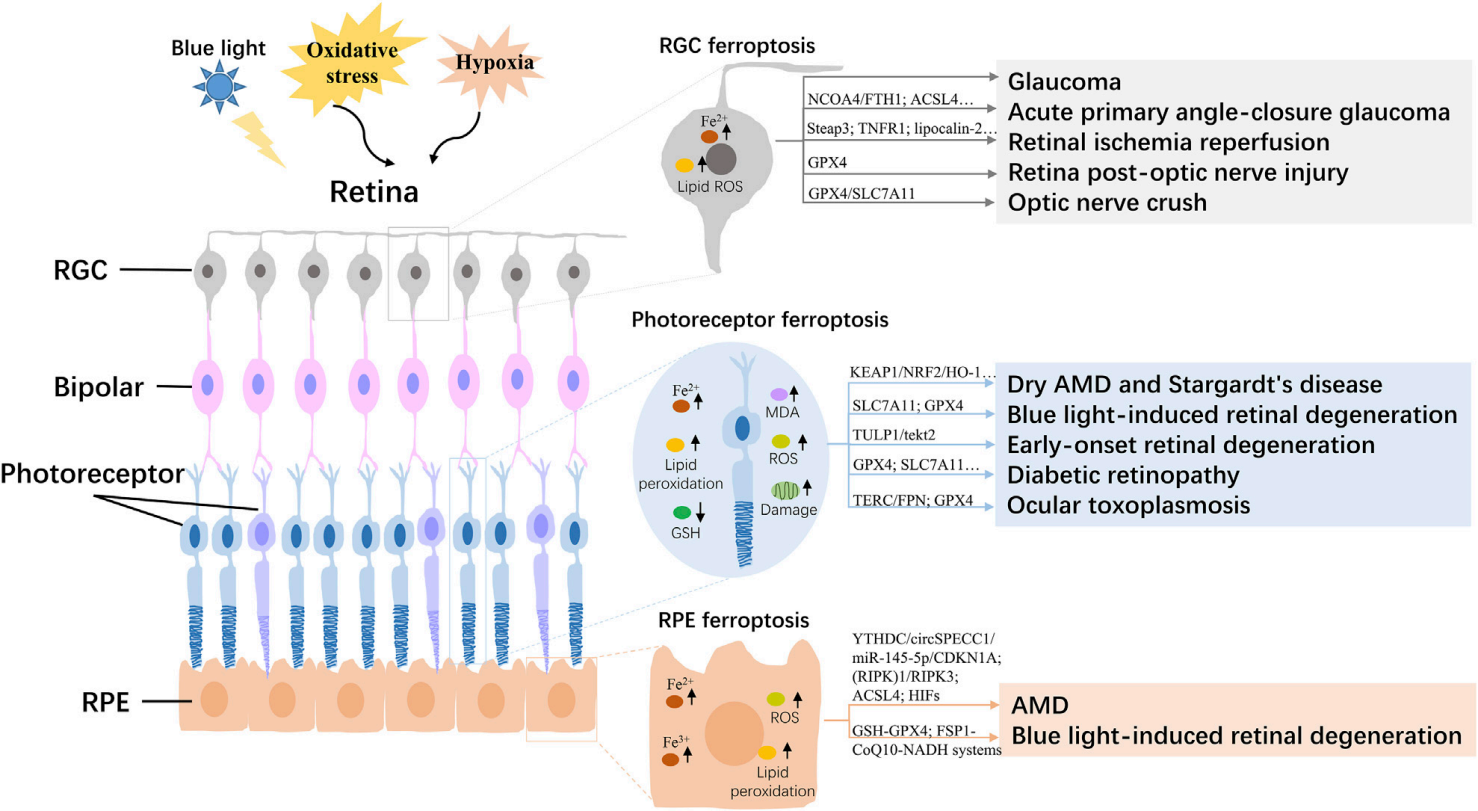
Overview of the regulatory mechanism of ferroptosis in the retina. AMD, age-related macular degeneration; RGC, retinal ganglion cell; RPE, Retinal pigment epithelial; ROS, reactive oxygen species; GSH, glutathione; MDA, malondialdehyde.

The retina is mainly composed of the inside out of retinal pigment epithelial cells (RPE), photoreceptor cells, horizontal cells, bipolar cells, node cells, amacrine cells, and retinal ganglion cells (RGC), as the key part of the vision with the functions that captures light and passes it to the brain (Hoon et al., 2014). Ferroptosis of retinal cells, such as RPE, photoreceptor, and RGC, has been reported to be involved in the progression of age-related/inherited macular degeneration, blue light-induced retinal degeneration, glaucoma, DR, and retinal damage caused by retinal ischemia-reperfusion (I/R) (Figure 1; Table 1).

**TABLE 1:** Pathological role of ferroptosis in retinal diseases.

Disease/Symptom	Cell types	Ferroptosis feature	Molecular mechanism	Function	Ref
AMD	RPE	Increased lipid peroxidation and decreased cell viability	ACSL4	Oxidative stress and docosahexaenoic acid injury lead to ferroptosis in RPE	Neiteler et al. (2023)
		Elevation of Fe^3+^ levels and promoting the Fenton reaction	HIFs	Aggravates ferroptosis in RPE cells treated by hypoxia	Henning et al. (2022)
		OS-induced ferroptosis, depolarization, and irregular lipid metabolism	YTHDC/circSPECC1/miR-145-5p/CDKN1A	Induces visual impairments and RPE anomalies and interrupts retinal homeostasis	Chen et al. (2022b)
		Lipid ROS accumulation	RIPK1/RIPK3	Induces RPE cell ferroptosis	Tong et al. (2023)
Dry AMD and Stargardt’s disease	Photoreceptor	Fe^2+^ overload, tripeptide GSH depletion, damaged mitochondria and lipid peroxidation	ACSL4; MT-CO2; system Xc (−)	Photoreceptor ferroptosis elicited by atRAL	Chen et al. (2021)
		Elevation of Fe^2+^ levels, ROS overproduction and ROS-mediated lipid peroxidation	KEAP1/NFE2L2/HMOX1	Photoreceptor ferroptosis elicited by atRAL	Chen et al. (2023)
Retinal degeneration	Photoreceptor	Fe^2+^ induced iron accumulation, oxidative damage and autofluorescence	GPX4; MT-CO2	Resulting in photoreceptor death and retinal RPE autofluorescence	Shu et al. (2020)
Blue light-induced retinal degeneration	Retina	Upregulation of ferroptosis-related genes	Jun; Stat3; Hmox1; Atf3; Hspa5; Ripk1	Causes retinal degeneration by inducing ferroptosis	Lei et al. (2023)
	Photoreceptor	Increased iron content and intracellular MDA content, decreased level of GSH and smaller mitochondria	SLC7A11; GPX4	Promotes ferroptosis in 661W cells	Tang et al. (2021b)
	ARPE-19	Fe^2+^ burst, lipid peroxidation, and decreased cell viability	GSH-GPX4; AIFM2-CoQ10-NADH systems	Causes retinal damage and degeneration by inducing ferroptosis	Li et al. (2023b)
Early-onset retinal degeneration	Retina	Upregulation of ferroptosis related genes, the shrinkage of mitochondria, reduction or disappearance of mitochondria cristae, and the iron and lipid droplet deposition	TULP1/TEKT2	Increases the death of photoreceptors via ferroptosis	Jia et al. (2022)
Glaucoma	RGC	Increased iron in the retina of glaucoma patient’s eye as compared to non-glaucomatous eyes	—	RGC death induced by dysregulation of iron homeostasis	Youale et al. (2022)
Acute primary angle-closure glaucoma	RGC	Abnormal accumulation of ferrous iron and pro-ferroptotic factors, and a depletion of anti-ferroptotic factors	NCOA4/FTH1; ACSL4; GPX4	Disturbance of iron metabolism and ferroptosis in RGCs during glaucoma	Yao et al. (2023)
Retinal ischemia reperfusion	RGC	Generation of glutamate and ferrous iron	Steap3	Retinal damage after ischemia-reperfusion	Dvoriantchikova et al. (2022)
		Significant changes in the signaling cascades regulating ferrous iron (Fe^2+^) metabolism	TNFRSF1A; FAS	Leading to retinal damage after ischemia-reperfusion	Dvoriantchikova et al. (2023)
		Upregulation of lipocalin-2 promoted ferroptosis signaling	lipocalin-2	Involved in RGC death and visual impairment	Mei et al. (2022)
		Accelerated lipid peroxidation	NCOA4; FTH; FTL	Regulating ferritinophagy of RGCs during retinal ischemia reperfusion	Dong et al. (2023)
Retina post-optic nerve injury	RGC	Upregulated of ferroptosis marker (4-hydroxynonenal)	GPX4	Cell death involved in the RGC death post- optic nerve injury	Yao et al. (2022)
NMDA-induced neuronal degeneration	Retina	Upregulation of ACSL3 and PRNP proteins	ACSL3; PRNP	Links to pathological cell death in the retina with NMDA insult	Suo et al. (2022)
Optic nerve crush	RGC	Downregulation of GPX4 and SLC7A11, and increased lipid peroxide and iron levels	GPX4/SLC7A11	RGC ferroptosis	Guo et al. (2022)
Diabetic retinopathy	Retina	Differentially expression of ferroptosis-related genes	HMOX1; PTGS2	Participates in the occurrence and development of diabetic retinopathy	Huang et al. (2023)
	HRCECs	Accumulation of lipid-ROS, MDA, and decreased SOD and GSH-Px levels	TRIM46/GPX4	High glucose-induced ferroptosis and cell growth inhibition	Zhang et al. (2021)
	661W and photoreceptor	Enhanced levels of iron, ROS, and MDA and reduced GSH concentration	GPX4; SLC7A11; ACSL4, FTH1; NCOA4	Photoreceptor degeneration in the development of the early stages of diabetic retinopathy	Gao et al. (2023)
Air pollution associated retinal vascular diseases	HRMECs	Iron overload and excessive lipid oxidation	MT-CO2; GPX4; FTH1	PM2.5-induced HRMEC cytotoxicity and dysfunction	Gu et al. (2022)
Oxygen-induced retinopathy	Retina	Hypoxic cell injury/death (ferroptosis)	HDDC3/MESH1	Early pathological vascular hyperpermeability	Vahatupa et al. (2023)
Ocular toxoplasmosis	Photoreceptor	Accumulation of iron in the neurosensory retina, increased lipid peroxidation, reduction of GPX4 expression and mitochondrial deformity	TERC/SLC40A1; GPX4	Induction of retinochoroiditis during ocular toxoplasmosis	Yamada et al. (2023)
L-selenomethionine-induced ocular defects	Zebrafish embryos	Disrupted mitochondrial morphology, elevated ROS-induced oxidative stress, apoptosis and ferroptosis	—	A key role in selenium-induced defects of embryonic eye development	Gao et al. (2022)
Retinopathy in fatty acid oxidation disorders	Photoreceptor	Disrupted energy homeostasis in the retina, leading to lipid droplet deposition and promoting ferroptosis	CPT1	Photoreceptor degeneration and visual impairments in cpt1a-MO zebrafish	Ulhaq et al. (2023)

Notes: AMD, age-related macular degeneration; RGC, retinal ganglion cell; RPE, retinal pigment epithelial; OS, oxidative stress; ROS, reactive oxygen species; GSH, glutathione; atRAL, all-transretinal; MDA, malondialdehyde; NMDA, N-methyl-D-aspartate; HRCECs, human retinal capillary endothelial cells; HRMECs, human retinal microvascular endothelial cells; Ref, reference.

### 2.1 Age-related/inherited macular degeneration

The RPE performs many crucial functions, such as phagocytosis, barrier, nutrient transportation, oxidative stress resistance, visual cycle maintenance and factors production, and is essential for the survival and function of the retina (Wang S. et al., 2023). RPE dysfunction and atrophy cause various retinal diseases including AMD. Recent studies showed that ferroptosis in RPE cells participates in the pathogenesis of AMD through multiple molecular regulatory mechanisms (Chen et al., 2021; Chen X. et al., 2022; Henning et al., 2022; Chen et al., 2023; Neiteler et al., 2023; Tong et al., 2023) and is a potential therapeutic target for AMD.

Oxidative stress and hypoxia-induced RPE cell death have long been considered to play crucial roles in the initiation and progression of AMD, and ferroptosis was identified as one of the main cell death mechanisms of oxidative stress (Henning et al., 2022; Neiteler et al., 2023). Iron accumulation and altered iron metabolism are characteristic features of AMD retinas and RPE cells (Henning et al., 2022). Oxidative stress and docosahexaenoic acid injury-induced downregulation of ferroptosis regulator acyl-CoA synthetase long-chain family member 4 (ACSL4), increased lipid peroxidation and decreased cell viability, and lead to ferroptosis in RPE (Neiteler et al., 2023). Hypoxia aggravates ferroptosis in RPE cells by elevating Fe^3+^ levels, and promoting the Fenton reaction (Henning et al., 2022). In RPE of AMD patients, the expression of circSPECC1 is downregulated, and circSPECC1 insufficiency leads to oxidative stress-induced ferroptosis, depolarization, and irregular lipid metabolism via circSPECC1/miR-145-5p/CDKN1A axis, and then induces visual impairments and RPE anomalies and interrupts retinal homeostasis (Chen X. et al., 2022). In addition, RAS-selective lethal three induced-RPE ferroptosis shows the features of receptor-interacting protein kinase (RIPK)1/RIPK3 activation and lipid ROS accumulation (Tong et al., 2023).

Dysfunction of photoreceptors in dry AMD and Stargardt disease (SD) is associated with defects of atRAL clearance (Chen et al., 2021). The overload of atRAL lead to Fe^2+^ overload, tripeptide glutathione (GSH) depletion, damaged mitochondria and lipid peroxidation in photoreceptors via ACSL4 elevation, system Xc(−) inhibition and MT-CO2 activation, resulting in photoreceptor ferroptosis and dysfunction (Chen et al., 2021). HMOX1 is a key factor that regulates atRAL-induced ferroptosis in photoreceptors via KEAP1/NFE2L2/HMOX1 axis, and its inhibition may be a novel therapeutic strategy for the treatment of dry AMD and SD (Chen et al., 2023).

### 2.2 Retinal degeneration induced by blue light or others

Retinal photoreceptors, mainly including rod and cone cells, are a special type of neuroepithelial cells with light transduction ability with the function of converting light signals into neural signals (Zhao and Peng, 2021). Photoreceptor degeneration and death, as one of the main characteristics of retinal degeneration diseases, lead to severe visual impairment (Wang et al., 2024).

Ferroptosis is implicated in the pathogenesis of retinal degeneration. Fe^2+^ induced iron accumulation, oxidative damage and autofluorescence of photoreceptors, resulting in photoreceptor death and RPE autofluorescence in mouse models (Shu et al., 2020). Light damage is a widely used model for retinal degeneration. In the light damage retina of mice models, ferroptosis genes, such as *Jun*, *Stat3*, *Hmox1*, *Atf3*, *Hspa5* and *Ripk1*, were significantly upregulated, which suggests the important role of ferroptosis in retinal degeneration (Lei et al., 2023). The photoreceptor cells showed pro-ferroptotic changes after light exposure, including increased iron content and intracellular malondialdehyde (MDA) content, decreased level of GSH, smaller mitochondria and decreased protein expression of SLC7A11 and GPX4, resulting in decreased cell viability *in vitro* (Tang W. et al., 2021). In addition, light also triggers ROS and Fe^2+^ burst, lipid peroxidation, and decreased cell viability of RPE cell lines, resulting in retinal damage and degeneration by inducing ferroptosis via GSH-GPX4 and AIFM2-CoQ10-NADH systems (Li X. et al., 2023). Through TULP1 knockout models in zebrafish, scientists found that TULP1 deficiency causes early-onset retinal degeneration through up-regulating ferroptosis-related genes, increasing the shrinkage of mitochondria, iron and lipid droplet deposition and reducing mitochondria cristae, and increasing the death of photoreceptors via ferroptosis (Jia et al., 2022).

### 2.3 Glaucoma

RGC is located in the innermost layer of the retina, composed of multipolar ganglion cells, and its dendrites are mainly connected to bipolar cells (Masland, 2012). Its axons extend to the optic nerve nipple, pass through the sieve plate, and form the optic nerve, playing a role in transmitting visual signals to the brain (Parisi et al., 2018). RGC degeneration is a common cause of glaucoma and optic neuropathy, which is the main cause of irreversible blindness and visual impairment (Ju et al., 2023).

Pathological high intraocular pressure (pH-IOP) is the main cause of visual impairment in glaucoma patients, which can lead to RGC death and permanent visual impairment (Yao et al., 2023). Research has found that the iron staining is increased in the retina of a glaucoma patient’s eye as compared to non-glaucomatous eyes (Youale et al., 2022). The levels of ferric iron in the peripheral serum of patients with acute primary angle closure glaucoma are significantly higher than the average population, indicating that iron homeostasis is involved in regulating the damage of RGCs under pH IOP (Yao et al., 2023). pH IOP leads to abnormal accumulation of ferrous iron and pro-ferroptotic factors including lipid peroxidation and ACLS4, and depletion of anti-ferroptotic factors, such as GSH, GPX4, and nicotinamide adenine dinucleotide phosphate, in RGC cells and retinas via NCOA4/FTH1 axis, thus disrupting iron metabolism and leading to ferroptosis in RGCs during glaucoma (Yao et al., 2023).

### 2.4 Retinal ischemia-reperfusion

Retinal I/R injury, as a common cause of irreversible visual impairment in clinical can lead to RGC death and is implicated in the pathological process of glaucoma, retinal artery occlusion and DR and other eye diseases. Multiple research results indicate that RGC ferroptosis is involved in the process of retinal damage triggered by I/R (Dvoriantchikova et al., 2022; Mei et al., 2022; Dong et al., 2023; Dvoriantchikova et al., 2023).

RNA-seq data analysis result of a retinal I/R mouse model showed RGC ferroptosis contributes simultaneously to retinal damage after I/R, through Steap3 promoting the glutamate, and ferrous iron generation, which trigger ferroptosis in ischemic RGC (Dvoriantchikova et al., 2022). RGCs isolated from retinas 24 h after IR show that the signaling cascades regulating ferrous iron (Fe^2+^) metabolism, such as TNFRSF1A and FAS, undergo significant changes, which causes retinal damage after I/R (Dvoriantchikova et al., 2023). After IOP-induced retinal I/R injury, lipocalin-2 transgenic mice showed more aggravated RGC death and visual impairment compared with wild-type mice (Mei et al., 2022). Lipocalin-2 plays an important role in RGC death and visual impairment via strongly promoting ferroptosis signaling (Mei et al., 2022). NCOA4, a cargo receptor for ferritinophagy, is upregulated and accelerates lipid peroxidation in retinal tissues, and regulates ferritinophagy of RGCs during retinal I/R (Dong et al., 2023).

### 2.5 Optic nerve injury

Irreversible visual impairment and blindness occur as a result of optic nerve injury, with RGC death being a crucial pathological initiator. Following optic nerve injury, ferroptosis emerges as a significant mode of cell death for RGCs (Yao et al., 2022). Following the early reduction of GPX4, the upregulation of the ferroptosis marker (4-hydroxynonenal) was observed in mouse retina between Day 7 and Day 14 post-ON injury, indicating the ferroptosis in RGC (Yao et al., 2022). Ferroptosis is also linked to pathological cell death in the retina with N-methyl-D-aspartate (NMDA) insult (Suo et al., 2022). The ACSL3 and PRNP proteins were upregulated in the NMDA-damaged retina and connected with ferroptosis (Suo et al., 2022). For the optic nerve crush rat models, the RGC shows a reduction of GPX4 and SLC7A11 expression, increased membrane density, reduced mitochondrial cristae, and increased lipid peroxide and iron levels, indicating RGC ferroptosis after optic nerve crush (Guo et al., 2022).

### 2.6 Diabetic retinopathy

DR is one of the most common microvascular complications of diabetes, which is a series of fundus diseases caused by retinal microvascular leakage and occlusion caused by chronic progressive diabetes. Ferroptosis is involved in the occurrence and development of DR. RNA sequencing results showed that the expression of ferroptosis-related genes in DR retinas changed significantly, and *HMOX1* and *PTGS2* genes were hub genes of differentially expressed ferroptosis-related genes (Huang et al., 2023). High glucose induces accumulation of lipid-ROS and MDA, and decrease of superoxide dismutase and GSH levels in human retinal capillary endothelial cells (HRCECs), then leading to ferroptosis and cell growth inhibition via TRIM46/GPX4 ubiquitination axis, and results in dysfunction in HRCECs (Zhang et al., 2021). In addition, the photoreceptor cells in diabetic mice also showed enhanced levels of iron, ROS, and MDA and reduced GSH concentration with downregulation of GPX4 and SLC7A11, and upregulation of ACSL4, FTH1 and NCOA4, which suggested that ferroptosis is also involved in the pathogenesis of photoreceptor degeneration in the development of the early stages of DR (Gao et al., 2023).

### 2.7 Other retinal vascular diseases

Ferroptosis is also involved in the pathogenesis of other retinal vascular diseases, such as air pollution associated retinal vascular diseases (Gu et al., 2022) and oxygen-induced retinopathy (Vahatupa et al., 2023). Airborne fine particulate matter (PM2.5) exposure induces iron overload and excessive lipid oxidation with significantly altered expression of ferroptosis-related genes (MT-CO2, GPX1 and FTH1) in human retinal microvascular endothelial cells, suggesting that ferroptosis plays a crucial role in PM2.5-induced HRMEC cytotoxicity and dysfunction, and is a potential precautionary target in air pollution associated retinal vascular diseases (Gu et al., 2022). Besides, in the retina of Rras knockout mice, hypoxia can cause early pathological vascular hyperpermeability via hypoxic cell injury/death (ferroptosis), indicating an important role of ferroptosis in ischemic retinal diseases (Vahatupa et al., 2023).

### 2.8 Ocular toxoplasmosis

Ocular toxoplasmosis (OT), as the main clinical manifestation of toxoplasmosis, seriously threatens the patient’s vision. The study found that the iron concentration in the vitreous of human OT patients decreased significantly, suggesting that ferroptosis is involved in the pathogenesis of OT (Yamada et al., 2023). The eyes after infecting toxoplasma gondii showed accumulation of iron in the neurosensory retina, and increased lipid peroxidation, reduction of GPX4 expression and mitochondrial deformity in the photoreceptor, suggesting that ferroptosis plays a crucial role in the induction of retinochoroiditis during ocular toxoplasmosis (Yamada et al., 2023).

### 2.9 Other ocular defects in zebrafish

In zebrafish embryos, excessive selenium disrupted mitochondrial morphology and elevated ROS-induced oxidative stress, apoptosis and ferroptosis (Gao et al., 2022). Ferroptosis plays a key role in l-selenomethionine-induced defects of embryonic eye development (Gao et al., 2022). The deficiency of carnitine palmitoyltransferase I (CPT1) was reported to disrupt energy homeostasis in the retina, leading to lipid droplet deposition and promoting ferroptosis in zebrafish models, which is attributed to the photoreceptor degeneration and visual impairments of fatty acid oxidation disorders (Ulhaq et al., 2023).

## 3 Pharmacological agents targeting ferroptosis in retinal diseases

**FIGURE 2:**
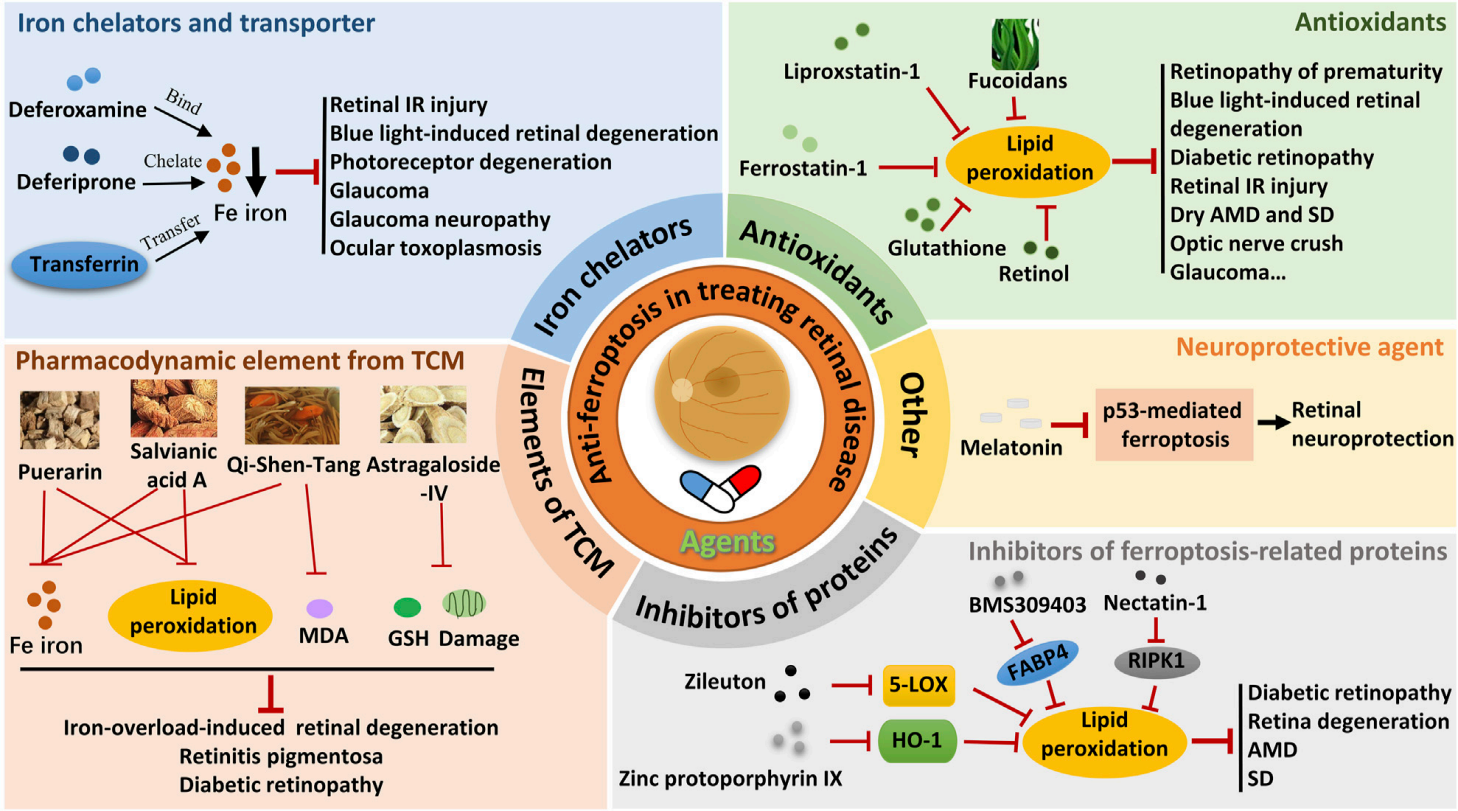
Therapeutic strategies targeting ferroptosis in retinal diseases. IR, ischemia-reperfusion; MDA, malondialdehyde; AMD, age-related macular degeneration; SD, Stargardt’s disease; GSH, glutathione; TCM, traditional Chinese medicine.

The critical role of retinal cell ferroptosis in various retinal diseases suggests a potential therapeutic role of ferroptosis inhibitors in related diseases. Ferroptosis inhibitors mainly act by reducing free iron, eliminating free radicals, and inhibiting lipid oxidation. Nearly 20 agents and extracts, including iron chelators and transporter, antioxidants, pharmacodynamic element from traditional Chinese medicine (TCM), ferroptosis-related protein inhibitors, and neuroprotective agent that inhibit ferroptosis have been reported to have a remissioning effect on retinal disease in animal models (Table 2; Figure 2).

**TABLE 2:** Pharmacological agents targeting ferroptosis in retinal diseases.

Molecule/Drug	Disease/Symptom	Effect on ferroptosis	Molecular mechanism	Function	Ref
Iron chelators and transporter					
Deferoxamine	Retinal IR injury	Inhibition of hemochromatosis, initiation of transferrin, degradation of ferritin and activating the antioxidant capacity	Xc-GSH-GPX4	Protects the structural and functional soundness of the retina by inhibiting ferroptosis	Wang et al. (2023b)
	Blue light-induced retinal degeneration	Reduced intracellular Fe^2+^ level, ROS burst and lipid peroxidation accumulation	GSH-GPX4; AIFM2-CoQ10-NADH	Improves blue light-triggered lipid peroxidation and cell death in ARPE-19 cells	Li et al. (2023b)
	Photoreceptor degeneration	Decreased Fe^2+^, total iron levels and LDH levels	MT-CO2; ACSL4	Protectes photoreceptor cells from ferroptosis caused by atRAL	Chen et al. (2021)
Deferiprone	Glaucoma	Chelating iron and ameliorating ph-IOP induced retinal ferroptosis	GPX4; ACSL4	Inhibits ferroptosis and protects RGCs from ph-IOP injury	Yao et al. (2023)
	Ocular toxoplasmosis	Reducing the iron uptake	—	Ameliorates toxoplasma-induced retinochoroiditis by reducing retinal inflammation	Yamada et al. (2023)
	Retinal IR injury	Suppressing ferritin degradation	—	Protects mouse retinas against IR stress	Dong et al. (2023)
Transferrin	Glaucoma neuropathy	Preventing iron overload and reducing iron-induced oxidative stress	—	Protectes RGCs from apoptosis, ferroptosis and necrosis	Youale et al. (2022)
Antioxidants					
Ferrostatin-1	Retinopathy of prematurity	Reduced lipid peroxidation and reversed the change of ferroptosis marker in retina	SLC7A11/GPX4; TFRC/FTH1	Attenuates pathological angiogenesis in oxygen-induced retinopathy via inhibition of ferroptosis	Liu et al. (2023a)
	Blue light-induced retinal degeneration	Reduced intracellular Fe^2+^ level, ROS burst and lipid peroxidation accumulation	GSH-GPX4; AIFM2-CoQ10-NADH	Alleviated retinal oxidative stress and degeneration in rats	Li et al. (2023b)
		Reduced levels of MDA and rescued cell death	SLC7A11; GPX4	Protects against light-induced retinal degeneration	Tang et al. (2021b)
	Diabetic retinopathy	Ameliorated the enhanced levels of iron, ROS, and MDA and reduced GSH concentration cuased by high-glucose	GPX4; SLC7A11; ACSL4, FTH1; NCOA4	Ameliorates photoreceptor degeneration in diabetic mice	Gao et al. (2023)
		Inhibited ferroptosis of RPE cells	—	Decreases RPE cell dead rate induced by high-glucose	Tang et al. (2022)
	Retinal IR injury	Suppressed ferritin degradation	—	Protects mouse retinas against IR stress	Dong et al. (2023)
		Inhibit lipid peroxidation	TF; SLC7A11; VDAC; GPX4; AIFM2; ACSL4	Marked RGC protection against IR	Qin et al. (2022)
	Dry AMD and SD	Relieved HMOX1 activation	HMOX1	Repress HMOX1-mediated ferroptosis	Chen et al. (2023)
	Photoreceptor degeneration dry AMD and SD	Inhibiting lipid peroxidation, and decreased LDH levels	MT-CO2; ACSL4	Protected photoreceptor cells from ferroptosis caused by atRAL, and inhibited photoreceptor ferroptosis in light-exposed Abca4(−/−)Rdh8(−/−)mice	Chen et al. (2021)
	Optic nerve crush and microbead-induced glaucoma	Prevented the production of mitochondrial lipid peroxides, the impairment of mitochondrial ATP production	-	Promoted RGC survival and preserved retinal function in mouse models	Guo et al. (2022)
Liproxstatin-1	Retinal IR	Inhibit ferroptosis	GPX4	Ameliorates RGC death and visual impairment	Mei et al. (2022)
GSH	Photoreceptor degeneration	GSH supplement	SLC7A11; MT-CO2; ACSL4	Protected photoreceptor cells from ferroptosis caused by atRAL.	Chen et al. (2021)
Fucoidans	Erastin-induced cell death in ARPE-19	Against oxidative stress and ferroptosis	GPX4	Against oxidative stress and ferroptosis of ARPE-19	Doerschmann et al. (2021)
Retinol, atRAL and atRA	Ferroptosis implicated dieases	Directly interdict lipid radicals in ferroptosis	—	Inhibit ferroptosis in neuronal and nonneuronal cell lines	Jakaria et al. (2023)
Pharmacodynamic element from traditional Chinese medicine					
Puerarin	Iron-overload-induced retinal degeneration	Decreasing excessive iron and lowering lipid peroxidation	NFE2L2/SLC7A11/GPX4/HMOX1; TFRC/SLC40A1; MT-CO2	Ameliorates retinal pathology, increases retinal cell viability, and protects against ferroptotic changes	Song et al. (2024)
Salvianic acid A	Iron-overload-induced retinal degeneration	Decreasing iron accumulation and relieved lipid peroxidation	ACSL4; SLC7A11/GPX4; TERC/FT/SLC40A1	Reduces mitochondrial dysfunction, againsts retinal iron overload by inhibition of ferroptosis, and alleviates retinal injury	Zhao et al. (2023)
Fructus Lycii and Salvia miltiorrhiza Bunge extract	Retinitis pigmentosa	Inhibit ferroptosis of photoreceptors	TP53; SLC7A11; GPX4	Prevents oxidative stress-induced photoreceptor ferroptosis and alleviates retinitis pigmentosa	Yang et al. (2023)
Qi-Shen-Tang	Retinitis pigmentosa	Iron and the production of MDA decreased	NFE2L2/GPX4	Inhibits ferroptosis, thereby reduces RP-induced damage to retinal tissue	Xiong et al. (2023)
Astragaloside-IV	Diabetic retinopathy	Decreased GSH content, mitochondria size and ridge	miR-138-5p/SIRT1/NFE2L2	Increases cellular antioxidant capacity to alleviate ferroptosis, resulting decreased RPE cell death	Tang et al. (2022)
Inhibitors of ferroptosis-related proteins					
Zileuton	Retina degeneration	Reduced the NaIO3-induced lipid peroxidation of RPE cells	ALOX5	Reduced degeneration of both the neuroretina and RPE monolayer cells	Lee et al. (2022)
Zinc protoporphyrin IX	Dry AMD and SD	Rescued photoreceptor cells against ferroptosis arising from atRAL overload	HMOX1	Effectively alleviates both photoreceptor degeneration and RPE atrophy in Abca4−/−Rdh8−/−mice in response to light exposure	Chen et al. (2023)
BMS309403	Diabetic retinopathy	Inhibited lipid peroxidation and oxidative stress	FABP4/PPARG	Alleviates lipid peroxidation and oxidative stress in DR	Fan et al. (2022)
Nectatin-1	AMD	Rescuing RPE ferroptosis	RIPK1/RIPK3	Inhibits RIPK1/RIPK3 activation and lipid ROS accumulation	Tong et al. (2023)
Neuroprotective agent					
Melatonin	Retinal IR injury	Inhibiting p53-mediated ferroptosis	TP53/SLC7A11/ALOX12	Alleviates retinal damage and RGC death and attenuates retinal IR-induced ferroptosis	Zhang et al. (2023)

Notes: IR, ischemia reperfusion; ROS, reactive oxygen species; LDH, lactate dehydrogenase; ph-IOP, pathologically high intraocular pressure; RGC, retinal ganglion cell; MDA, malondialdehyde; RPE, retinal pigment epithelial; AMD, age-related macular degeneration; SD, Stargardt’s disease; atRAL, all-transretinal; atRA, all-trans-retinoic acid; GSH, glutathione.

### 3.1 Iron chelators and transporter

#### 3.1.1 Deferoxamine

Deferoxamine (DFO) is an iron chelator (binds Fe (III) and many other metal cations) that is widely used to reduce the accumulation and deposition of iron in tissues. Through the inhibition of hemochromatosis, initiation of transferrin, degradation of ferritin and activation the antioxidant capacity, DFO protects the structural and functional soundness of the retina by inhibiting ferroptosis in the rat models of retinal I/R injury [33]. On the other hand, DFO improves blue light-triggered lipid peroxidation and cell death in ARPE-19 cells via reducing intracellular Fe^2+^ level, ROS burst and lipid peroxidation accumulation in the model of blue light-induced retinal damage and degeneration (Li X. et al., 2023). Furthermore, DFO protects photoreceptor cells from ferroptosis caused by atRAL by decreasing Fe^2+^ levels, total iron levels, and lactate dehydrogenase (LDH) levels (Chen et al., 2021). Via inhibition of ferroptosis, DFO has the potential to attenuate and against various retinal damage and degeneration and offer new strategies for treating ferroptosis-related retinal diseases.

#### 3.1.2 Deferiprone

Deferiprone (DFP) is a Food and Drug Administration (FDA) approved oral active iron chelator with brain permeability, cell permeability and skin permeability, which is currently used in the clinical treatment of iron overload. *In vivo* studies have demonstrated that the oral intake of DFP effectively binds to iron and improves the condition of retinal ferroptosis induced by ph-IOP (Yao et al., 2023). Furthermore, it inhibits ferroptosis and safeguards RGCs against ph-IOP-related damage (Yao et al., 2023). These findings offer valuable insights into the role of iron homeostasis and ferroptosis pathways in the understanding and treatment of glaucoma. Furthermore, the iron uptake was diminished by DFP while simultaneously improving the condition of retinochoroiditis caused by toxoplasma through the reduction of retinal inflammation (Yamada et al., 2023). This presents a promising therapeutic option for OT. In addition, it was reported that DFP inhibits the breakdown of ferritin, limits cell death and inflammation, and safeguards the retinas of mice from I/R stress (Dong et al., 2023).

#### 3.1.3 Transferrin

TF serves as an endogenous iron transporter that plays a crucial role in regulating iron levels within the eye. By preventing iron overload and reducing oxidative stress caused by iron, the intraocular delivery of TF has been found to have neuroprotective effects in various retinal degeneration models (Bigot et al., 2020). TF also has the ability to intervene in apoptosis, ferroptosis and necrosis associated with glaucoma pathogenesis, demonstrating its potential to shield RGCs from elevated intraocular pressure (Youale et al., 2022). These collective findings suggest that TF could be a promising treatment for glaucomatous neuropathy.

### 3.2 Antioxidants

#### 3.2.1 Ferrostatin-1

Ferrostatin-1 (Fer-1, C_15_H_22_N_2_O_2_), as an artificially synthesized antioxidantis, is a potent and selective inhibitor of ferroptosis. Via inhibition of ferroptosis, Fer-1 has the potential to attenuate and against various retinal damage and degeneration, and provides new potential strategies for the treatment of ferroptosis-related retinal diseases.

In a mouse model of oxygen-induced retinopathy, Fer-1 administration attenuates pathological angiogenesis in the retina via inhibition of ferroptosis, suggesting a promising target for retinopathy of prematurity therapy (Liu C.-Q. et al., 2023). Fer-1 alleviates ferroptosis and protects the retina against light-induced retinal degeneration in rats (Tang W. et al., 2021; Li X. et al., 2023). Via inhibition of ferroptosis, Fer-1 ameliorates photoreceptor degeneration and decreases the RPE cell death rate caused by high glucose, and has also shown potential therapeutic effects in DR (Tang et al., 2022; Gao et al., 2023). In mouse models of retinal I/R, Fer-1 administration suppressed ferritin degradation and lipid peroxidation, restrained apoptosis and inflammation of the retina, inhibited RGC death, and protected mouse retinas against I/R stress (Qin et al., 2022; Dong et al., 2023). Fer-1 administered intraperitoneally also shows potential in treating dry AMD and SD by inhibiting atRAL-induced ferroptosis in photoreceptor and RPE cells of light-exposed Abca4(−/−)Rdh8(−/−)mice (Chen et al., 2021; Chen et al., 2023). Fer-1 demonstrated a substantial enhancement in the survival of RGC and the preservation of retinal function in mouse models of optic nerve crush and microbead-induced glaucoma (Guo et al., 2022). This compound holds the potential as a protective approach for mitigating RGC injuries in optic neuropathies.

#### 3.2.2 Liproxstatin-1

Liproxstatin-1 (C19H21ClN4) is a potent inhibitor of ferroptosis with antioxidant and anti-inflammatory effects, able to inhibit ferroptosis in the low nanomolar range. Liproxstatin-1 could significantly ameliorate RGC death and visual impairment in the pathogenesis of ischemic retinopathy, indicating its potential as a treatment for retinal IR (Mei et al., 2022).

#### 3.2.3 Glutathione

GSH is a cofactor of GPX4, a lipid peroxidase that inhibits ferroptosis. GSH supplement protects photoreceptor cells from ferroptosis caused by atRAL, and may provide a new strategy for protection against dry AMD and SD (Chen et al., 2021).

#### 3.2.4 Fucoidans

Fucoidans, derived from various algae species such as F. serratus, F. distichus subsp. evanescens, and Laminaria hyperborea, have been shown to possess protective properties against oxidative stress. Specifically, these fucoidans have been found to mitigate the reduction in protein levels of the antioxidant enzyme GPX4, offering partial protection against erastin-induced oxidative stress and ferroptosis in ARPE-19 cells (Doerschmann et al., 2021). These results indicate that fucoidans with protective qualities could potentially serve as therapeutic agents for conditions related to ARP ferroptosis. Further research is necessary to fully understand the beneficial effects of fucoidans on health and to elucidate the precise mechanisms underlying their antioxidative properties.

#### 3.2.5 Vitamin A metabolites

Vitamin A, also known as retinol, is a fat-soluble vitamin that serves as a precursor for various bioactive substances, including retinaldehyde (retinal) and different forms of retinoic acid (Jakaria et al., 2023). Retinol and its metabolites, such as atRAL and all-trans-retinoic acid, have the ability to prevent ferroptosis in both neuronal and nonneuronal cell types by directly blocking lipid radicals (Jakaria et al., 2023). These compounds could potentially be used as treatments for conditions associated with ferroptosis. It has been noted that excessive concentrations of retinoids can be toxic to retinal cells (Chen et al., 2021; Jakaria et al., 2023). As a result, establishing the correct dosage concentration is an important consideration that should be taken into account in upcoming clinical trials.

### 3.3 Pharmacodynamic elements from traditional Chinese medicine

#### 3.3.1 Puerarin

Puerarin (C21H20O9), a natural isoflavone derived from Pueraria lobata and utilized in traditional Chinese herbal medicine, has been proven to possess preventive properties against iron overload (Song et al., 2020). Its efficacy in mitigating iron overload-induced ferroptosis by decreasing excessive iron and lowering lipid peroxidation in the retina through an NFE2L2-mediated mechanism, ameliorates retinal pathology, increases retinal cell viability, protects against ferroptotic changes in mouse models, which highlights its potential as a novel approach in managing retinal degeneration (Song et al., 2024).

#### 3.3.2 Salvianic acid A

Salvianic acid A (C9H10O5) is a significant pharmacodynamic element present in the water-soluble components of Salvia miltiorrhiza herb. It was reported to possess antioxidative properties and act as an anti-inflammatory agent in cases of liver injury due to iron overload (Zhang Y. et al., 2020). Through Decreasing iron accumulation and relieving lipid peroxidation, salvianic acid A is effective in reducing mitochondrial dysfunction, inhibiting ferroptosis to combat retinal iron overload, and alleviating retinal damage in iron overload mouse models, and holds potential as an innovative treatment strategy for retinal degeneration caused by iron overload (Zhao et al., 2023).

#### 3.3.3 Fructus Lycii and Salvia mTCNiltiorrhiza bunge extract

Fructus Lycii (Gouqizi) and Salvia miltiorrhiza Bunge (Danshen) are commonly utilized in TCM for addressing deficiencies, improving blood circulation, or as daily nutritional supplements (Amagase et al., 2009; Shi et al., 2019). The use of extracts from Fructus Lycii and Salvia miltiorrhiza Bunge can help prevent oxidative stress-induced photoreceptor ferroptosis and reduce the severity of RP by inhibiting photoreceptor ferroptosis through the TP53/SLC7A11 pathway (Yang et al., 2023).

Furthermore, another investigation revealed that Qi-Shen-Tang, a TCM formula containing Gouqizi and Danshen in a 1:1 ratio, can suppress ferroptotic characteristics of the retina in mice with RP by blocking the NFE2L2/GPX4 signaling pathway (Xiong et al., 2023). This ultimately leads to a decrease in retinal tissue damage caused by RP. These findings suggest that these herbal extracts could be beneficial in clinical treatments for RP.

#### 3.3.4 Astragaloside-IV

Decoctions made from the roots of Astragalus membranaceus, commonly referred to as “Huangqi,” have been extensively utilized in TCM to address viral and bacterial infections as well as inflammation (Zhang J. et al., 2020). Astragaloside IV (AS-IV, C41H68O14), a significant component found in the aqueous extract of Astragalus membranaceus, is a cycloartane-type triterpene glycoside with proven biological properties (Zhang J. et al., 2020). Laboratory experiments have revealed that AS-IV reduces GSH levels, alters the size and structure of mitochondria in RPE cells, enhances cellular antioxidant capabilities to mitigate ferroptosis, and ultimately decreases high glucose-induced RPE cell death through the miR-138-5p/SIRT1/NFE2L2 pathway (Tang et al., 2022). The findings suggest that AS-IV can alleviate ferroptosis triggered by high glucose in RPE cells, indicating its potential as a treatment for diabetic retinopathy.

### 3.4 Inhibitors of ferroptosis-related proteins

#### 3.4.1 Lipoxygenase inhibitor

Lipoxygenases (LOXs) are enzymes that metabolize arachidonic acid and polyunsaturated fatty acids, leading to lipid peroxidation and ferroptosis induction (Stockwell et al., 2017). Zileuton, a specific inhibitor of ALOX5, decreased NaIO3-induced lipid peroxidation in RPE cells, as well as the degeneration of neuroretina and RPE monolayer cells (Lee et al., 2022). This suggests that Zileuton could be a valuable strategy in managing ROS and ferroptosis-induced damage, which contribute to degeneration in retinal diseases.

#### 3.4.2 Zinc protoporphyrin IX

HMOX1 plays a crucial role in controlling atRAL-induced ferroptosis in photoreceptors and the RPE (Chen et al., 2023). Zinc protoporphyrin IX, acting as an inhibitor of HMOX1, protected photoreceptor cells from ferroptosis caused by atRAL overload (Chen et al., 2023). This intervention effectively reduced both photoreceptor degeneration and RPE atrophy in Abca4−/−Rdh8−/−mice exposed to light, indicating the potential of zinc protoporphyrin IX for treating dry AMD and SD (Chen et al., 2023).

#### 3.4.3 BMS309403

BMS-309403 (C31H26N2O3) demonstrates strong inhibitory effects on adipocyte fatty acid binding protein (FABP) and is orally active and selective. Both *in vitro* and *in vivo* studies have confirmed that treatment with BMS309403 effectively suppresses lipid peroxidation, oxidative stress, and ferroptosis in DR by modulating FABP4/PPARG-mediated ferroptosis (Fan et al., 2022). These findings suggest that BMS-309403 holds promise as a therapeutic option for retinal DR.

#### 3.4.4 Necrostatin-1

Necrostatin-1 (C13H13N3OS) is an inhibitor of receptor-interacting protein kinase 1 (RIPK1). As the RIPK1-targeted inhibitor of necroptosis, necrostatin-1 could both rescue the ferroptosis and necroptosis of RPE cells via inhibiting RIPK1/RIPK3 activation and lipid ROS accumulation (Tong et al., 2023). This compound shows promise as a potential treatment for geographic atrophy in AMD.

### 3.5 Neuroprotective agent

Acetyl–5–methoxytryptamine, also known as melatonin (C13H16N2O2), is a hormone that is secreted by the pineal gland in the brain, and shows great potential as a neuroprotective agent (Su et al., 2015). A recent study has shown that melatonin has the ability to alleviate retinal damage and RGC death and protect against retinal I/R injury by inhibiting TP53-mediated ferroptosis (Zhang et al., 2023). This finding indicates that melatonin is a specific ferroptosis inhibitor that can defer damage to the retina, making it a promising therapeutic agent for retinal neuroprotection.

## 4 Challenges for the application of ferroptosis targeting agents

### 4.1 Approved ferroptosis targeting agents

Given the novel role of ferroptosis in the pathogenesis of various retinal diseases, ferroptosis is a promising therapeutic target. Currently, a limited number of iron chelators have received approval from the FDA or are undergoing clinical trials for conditions such as iron overload-related diseases, organ damage, and transplants (Ballas et al., 2018). DFO was the first iron chelator to be approved for subcutaneous or intravenous administration, with treatment lasting up to 10–12 h for chronic iron overload caused by blood transfusions. Exjade (deferasirox) is the first oral iron chelator approved by the FDA. Initially indicated for chronic transfusion-induced iron overload in children aged two and above, it was later approved in 2013 for patients aged 10 and older with non-transfusion-dependent thalassemia syndrome, a condition characterized by chronic iron overload. Additionally, reduced glutathione eye drops (ATC (Anatomical Therapeutic Chemical) code: V03AB32) have been given the green light for treating conditions like corneal ulcers, corneal epithelium peeling, keratitis, and early senile cataracts. Zileuton (DrugBank Accession Number: DB00744) has been approved for both the prophylaxis and chronic treatment of asthma in both adults and children. Puerarin (DrugBank Accession Number: DB12290) was approved for the treatment of cardiovascular diseases in China in 1993. Melatonin (ATC code: N05CH01) has been approved for use in autism spectrum disorders, Smith Magnis syndrome, as well as indications for falling asleep and sleep disorders. The FDA has approved the designation of EYS611 (a DNA plasmid encoding human transferrin) developed by Eyevensys as an orphan drug for the treatment of RP degeneration (Bigot et al., 2020). The application of most ferroptosis-targeting agents in retinal diseases is still in the preclinical stage of new drug development, and there are no clinical trials yet. This will be an important direction for future research.

### 4.2 Prospects of the pharmacodynamic element from TCM

Previous studies have shown that by targeting ferroptosis, some pharmacodynamic elements from TCM have certain effects or unique advantages in retinal diseases, and have potential research value for the efficacy of related retinopathy (Tang et al., 2022; Xiong et al., 2023; Yang et al., 2023; Zhao et al., 2023; Song et al., 2024). Through a well-designed clinical research approach, the advancement of research on treating retinal diseases with TCM can be facilitated. How to explore new TCM drugs that may have clinical value for retinal ferroptosis-related diseases based on TCM theory and clinical efficacy, and confirm the clinical efficacy based on reliable data, is a scientific problem that needs to be solved urgently in the research and development of new TCM drugs related to retinal diseases.

### 4.3 Limitations of ferroptosis targeting agents

The discovery of these potential therapeutic drugs provides some new perspectives and options for the treatment of retina-related diseases. However, some limitations may hinder the use of these potential drugs in clinical settings. With regard to the long-term use of iron chelators in patients with chronic diseases, these patients may experience specific side effects, such as rash, nausea and dizziness, diarrhea, vomiting, hearing and vision loss, abdominal pain, and increasing risk of infection (Mobarra et al., 2016; Entezari et al., 2022). The biological half-life of Fer-1 is only a few minutes, and the unsatisfactory pharmacokinetic and pharmacodynamic profiles are generally unacceptable for clinical applications (Chen J. et al., 2022). The oral administration of AS-IV results in a bioavailability of 7.4% in beagle dogs and 3.66% in rats (Zhang J. et al., 2020). This limited absolute bioavailability hinders its potential for oral use.

### 4.4 Questions need to be solved urgently

The therapeutic effects of ferroptosis inhibitors on retina-related diseases have been shown to be effective in preclinical trials. Future research should focus on the development of more potent ferroptosis inhibitors, improved drug properties, and ideally clinical testing related to retinal diseases.

With the development of modern ophthalmic examination technology and wide clinical application, fundus manifestations such as retinal vascular morphology and lesions can be visualized, which is helpful in observing the physiological and pathological changes during the progression of retinal diseases. In the clinical practice of ferroptosis, the correlation between retinal ferroptosis and visual acuity, fundus manifestations, objective imaging examinations of the eye, and biochemical examination indicators are explored to provide support for further accurate clinical positioning.

In addition, there are several key issues to be addressed for the further development of clinical development of ferroptosis-targeting drugs. What is the physiological role of ferroptosis in the eye? In the context of retinal disease, more evidence is needed to provide a complete view of the dynamic processes of ferroptosis that drive retinal degeneration. Can we identify reliable, sensitive biomarkers of ferroptosis in the retina? When should we use targeted ferroptosis drugs for specific pathological vision conditions, fundus manifestations, and/or stages of disease? What dosing modalities and regimens are used in the treatment of the retina without affecting other cells? What strategies can be implemented to enhance pharmacokinetic characteristics like oral bioavailability, half-life, and organ distribution to maximize the therapeutic efficacy of ferroptosis-targeted drugs? Addressing the key scientific questions discussed in this review will improve our understanding of the precise role of ferroptosis in various retinal diseases, thereby providing a new scientific basis for the prevention and treatment of retinal diseases with ferroptosis-targeted drugs.

## 5 Conclusion

Ferroptosis plays a vital role in the progression of various retinal diseases. Ferroptosis of retinal cells, such as RPE, photoreceptor, and RGC, has been reported to be involved in the progression of age-related/inherited macular degeneration, blue light-induced retinal degeneration, glaucoma, DR, and retinal damage caused by retinal IR via multiple molecular mechanisms. The analysis of the mechanism of retinal cell ferroptosis has brought new targeted strategies for the treatment of retinal vascular diseases, retinal degeneration, and retinal nerve diseases. Ferroptosis inhibitors mainly act by reducing free iron, eliminating free radicals, and inhibiting lipid oxidation. Nearly 20 agents and extracts, including iron chelators and transporter, antioxidants, pharmacodynamic elements from TCM, ferroptosis-related protein inhibitors, and neuroprotective agent have been reported to have a remissioning effect on retinal disease in animal models via inhibiting ferroptosis. However, just a limited number of iron chelators have received approval from the FDA or are undergoing clinical trials for conditions such as iron overload-related diseases, organ damage, and transplants. The application of most ferroptosis-targeting agents in retinal diseases is still in the preclinical stage of new drug development, and there are no clinical trials yet. This will be an important direction for future research. The therapeutic effects of ferroptosis inhibitors on retina-related diseases have been shown to be effective in preclinical trials. Future research should focus on the development of more potent ferroptosis inhibitors, improved drug properties, and ideally clinical testing related to retinal diseases.

## Glossary

AMD: age-related macular degeneration
DR: diabetic retinopathy
RP: retinitis pigmentosa
ROS: reactive oxygen species
RPE: retinal pigment epithelial cells
RGC: retinal ganglion cells
I/R: ischemia-reperfusion
ACSL4: acyl-CoA synthetase long-chain family member 4
SD: Stargardt disease
atRAL: all-transretinal
GSH: glutathione
MDA: malondialdehyde
pH IOP: pathological high intraocular pressure
NMDA: N-methyl-D-aspartate
HRCECs: human retinal capillary endothelial cells
PM2.5: airborne fine particulate matter
HRMECs: human retinal microvascular endothelial cells
OT: Ocular toxoplasmosis
CPT1: carnitine palmitoyltransferase I
TCM: traditional Chinese medicine
DFO: deferoxamine
LDH: lactate dehydrogenase
DFP: deferiprone
FDA: Food and Drug Administration
TF: transferrin
Fer-1: ferrostatin-1
atRA: all-trans-retinoic acid
AS-IV: astragaloside IV
LOX: lipoxygenase
RIPK: receptor-interacting protein kinase
ATC: Anatomical Therapeutic Chemical
